# Sense of coherence, off-job crafting, and mental well-being: A path of positive health development

**DOI:** 10.1093/heapro/daac159

**Published:** 2022-11-28

**Authors:** Martin Tušl, Jessica de Bloom, Georg F Bauer

**Affiliations:** Public and Organizational Health, Center of Salutogenesis, Epidemiology, Biostatistics and Prevention Institute, University of Zurich, Hirschengraben 84, 8001 Zurich, Switzerland; Department of Psychology, Tampere University, Tampere, Finland; Faculty of Economics and Business, University of Groningen, Groningen, The Netherlands; Public and Organizational Health, Center of Salutogenesis, Epidemiology, Biostatistics and Prevention Institute, University of Zurich, Hirschengraben 84, 8001 Zurich, Switzerland

**Keywords:** salutogenesis, sense of coherence, off-job crafting, mental well-being, cross-lagged panel model

## Abstract

Our study examines the core concept of salutogenesis—sense of coherence (SOC)—in relation to off-job crafting (OJC) and mental well-being (MWB). The original salutogenic model of health mainly addresses the protective function of SOC against adversity. In our study, we focus on the recently proposed path of positive health development that captures how SOC can contribute to positive health and thriving. We present and test our theoretical assumptions about one such path, examining OJC as a possible mechanism how SOC translates into MWB. We tested our proposed model using cross-lagged panel model with three waves of panel data from Swiss and German employees (*N* = 2217). We compared our model to alternative nested models and conducted indirect effect analysis to test longitudinal mediation. Our hypothesized model fitted the data well and we found support for our main hypothesis that OJC partially mediates the relationship between SOC and MWB. Further, we identified positive reciprocal relationships between SOC and MWB, as well as between OJC and MWB. Overall, our study provides evidence that OJC is one mechanism underlying the recently postulated path of positive health development in the salutogenic model. For health promotion, this implies that promoting SOC and OJC may help to strengthen individual well-being and lead to positive feedback loops that foster personal development and thriving.

## INTRODUCTION

### Background and aims

The salutogenic model of health is a well-established theory in health promotion (Antonovsky, 1979, 1987, 1996). Sense of coherence (SOC) and generalized resistance resources (GRRs) are its main pillars that explain how individuals cope with stressors and stay healthy. The original model is primarily concerned with how SOC helps individuals to cope with adversity. However, less attention has been paid to the role of SOC in enabling individuals to thrive and flourish. Following an earlier proposition (Bauer *et al.*, 2006), Bauer and colleagues (Bauer *et al.*, 2019) have suggested adding a path of positive health development to the original salutogenic model to illustrate an individual’s movement toward well-being and thriving and encouraged further research in this direction. The first aim of this study is to present our theoretical assumptions about one such path of positive health development building on relevant literature from salutogenesis and from the well-developed crafting research in occupational health psychology. The second aim of this study is to empirically test these assumptions by specifically examining off-job crafting (OJC) as a possible underlying mechanism of how SOC translates into positive health outcomes in terms of mental well-being (MWB).

### Salutogenic model of health and its key mechanisms

SOC is the main pillar of salutogenesis, and one of the most important personal resources related to health and well-being (Eriksson and Lindström, 2006; Eriksson, 2022). SOC is defined as a global orientation determining the degree to which life is perceived as comprehensible, manageable and meaningful (Antonovsky, 1979). Salutogenesis posits that life experiences shape SOC, which helps to mobilize GRRs on the individual level (e.g. attitudes, self-efficacy, knowledge) and environmental level (e.g. social support, material resources, cultural capital) to cope with stressors and manage tension, thus moving toward the ease-end of the ease-disease continuum (Antonovsky, 1979).

Although the focus of salutogenesis is on the origins of health, Antonovsky defined the positive end of the ease-disease continuum still in a negative way as the absence of pain and functional limitation (Mittelmark and Bull, 2013; Bauer *et al.*, 2019). The original salutogenic model assumes that life and health are continuously threatened by stressors: ‘We are all, always, in the dangerous river of life.’ (Antonovsky, 1996). Accordingly, SOC has mostly been studied as a protective factor in coping with stress and adversity (Surtees *et al.*, 2003; Wainwright *et al.*, 2007; Kouvonen *et al.*, 2008; Super *et al.*, 2014). However, less attention has been paid to the role of SOC in promoting positive health outcomes, such as personal development, thriving and meaning in life (Bauer *et al.*, 2019). The Ottawa Charter (World Health Organisation, 1986) defines health promotion as ‘the process of enabling the individual to increase control over, and to improve, their health. To reach a state of complete well-being, an individual or group must be able to identify and to realize aspirations, to satisfy needs and to change or cope with the environment’. Accordingly, individuals with a strong SOC may not only be more resilient when facing adversities but may also be better able to use their resources to move toward positive health, to thrive and to live an active and productive life (Bauer *et al.*, 2019). Crafting may be a prime, proactive strategy to shape one’s environment and to satisfy needs, thus moving toward positive health.

### Crafting as proactive behaviors to enhance resources

In contrast to reactive coping (Lazarus and Folkman, 1987), crafting refers to proactive, intentional and goal-oriented behaviors (de Bloom *et al.*, 2020). Originally, research on crafting focused predominantly on the work domain, generating rich empirical evidence on the benefits of so-called *job crafting* (Wrzesniewski and Dutton, 2001) for employees, such as increased work engagement (Petrou *et al.*, 2012), job performance (Tims *et al.*, 2015), job satisfaction (Tims *et al.*, 2013) and psychological capital (Vogt *et al.*, 2016). Over time, different theoretical models of crafting have been developed (see Tims *et al.*, 2022 for a review), and crafting research has expanded also to non-work domains (Petrou and Bakker, 2016; Demerouti *et al.*, 2019; de Bloom *et al.*, 2020; Pijpker *et al.*, 2022).

Our study is based on the recently developed identity-based integrative needs model of crafting which defines crafting as ‘substantial behavioral and cognitive changes individuals deliberately apply to their roles to satisfy their psychological needs’ [(de Bloom *et al.*, 2020), p. 4]. The model reflects that crafting occurs across different life domains (i.e. work or non-work domain), and is motivated by underlying psychological needs. The needs are defined by the DRAMMA model which stands for: detachment, relaxation, autonomy, mastery, meaning and affiliation (Newman *et al.*, 2014). The DRAMMA model integrates past needs theories and research areas, such as leisure sciences, recovery from work (Sonnentag and Fritz, 2007), flow (Csikszentmihalyi, 1990) and self-determination theory (Ryan and Deci, 2000), and explains how needs satisfaction translates into subjective well-being. Key characteristics of crafting are to perceive one’s needs imbalance, engage in activities that match own needs and aspirations, and thus enhance well-being via needs satisfaction (de Bloom *et al.*, 2020).

In our study, we focus on crafting in the non-work domain (i.e. OJC), which includes a broad spectrum of activities related to leisure, caring duties or volunteer work. This domain thus offers more flexibility and opportunities to engage in activities that are motivated by personal needs, goals and aspirations compared to the work domain which is more driven by organizational needs and goals. Moreover, leisure as a key target of OJC has been described as a core ingredient for personal development and overall well-being (Newman *et al.*, 2014; Loveday *et al.*, 2018).

### Relevance of the integrative needs model of crafting for salutogenesis

The needs-based model of crafting explains how individuals can build up resources and thrive via proactive needs satisfaction. Within the salutogenic model of health, we argue that crafting can represent one mechanism of positive health development. Complementary to Antonovky’s (Antonovky, 1979) explanation of how SOC helps in coping with stressors, we propose that SOC as a global orientation to life supports individuals in crafting because SOC helps one to (i) identify and understand one’s psychological needs; (ii) engage in crafting efforts to achieve needs satisfaction and (iii) use available and build-up new resources during the crafting process. In the present longitudinal study, we test this proposition by linking SOC and OJC with MWB as a positive health outcome. We use MWB as it is more closely related to key aspects of positive health such as thriving and personal development than physical health. Moreover, research has shown that mental health outcomes are more closely related to SOC (Eriksson, 2022) and to OJC (Kujanpää *et al.*, 2020) than physical health outcomes. Accordingly, we propose the following main hypothesis:

Hypothesis 1: OJC partially mediates the relationship between SOC and MWB.

In addition, we hypothesize and test each direct link underlying our proposed mediation model. Firstly, a strong SOC helps individuals to recognize stressors and mobilize GRRs to cope and manage tension (Antonovsky, 1987). Through the lens of positive health development (Bauer *et al.*, 2006, 2019), individuals with a strong SOC may be better able to identify their unfulfilled needs and engage in OJC using resources in their environment to address the perceived needs imbalance. Therefore, we expect a positive direct link between SOC and OJC.

Hypothesis 2: SOC has a positive direct effect on OJC.

Secondly, Antonovsky (Antonovsky, 1996) stated that one’s SOC is shaped by consistency of life experiences, underload–overload balance, and participation in socially valued decision-making. We argue that increasing the consistency of life experiences and contributing to an optimal underload–overload balance requires proactive shaping of one’s life in line with individual psychological needs (de Bloom *et al.*, 2020). Therefore, we expect a positive direct link between SOC and OJC.

Hypothesis 3: OJC has a positive direct effect on SOC.

Thirdly, satisfaction of the DRAMMA needs has been linked to various well-being outcomes such as vitality and life satisfaction (Kujanpää *et al.*, 2020). Therefore, we expect a positive direct link between OJC and MWB.

Hypothesis 4: OJC has a positive direct effect on MWB.

Finally, previous research has shown a positive link between SOC and mental health outcomes (Eriksson and Lindström, 2006; Eriksson, 2022). Therefore, we expect a positive direct link between SOC and MWB.

Hypothesis 5: SOC has a positive direct effect on MWB.

## METHODS

### Design

The study has a three-wave longitudinal panel design with a time interval of 3 months between the first and second waves and 5 months between the second and third waves. Specifically, we used a cross-lagged panel model (CLPM) to assess predictive relationships between our studied variables (Kuiper and Ryan, 2018). The measurement interval was chosen based on our assumption about the time necessary to detect a change in the studied variables. Dormann and Griffin have suggested that three to 6 months’ time lag is sufficient for most variables in occupational health research (Dormann and Griffin, 2015).

### Participants

We included the general working populations from Germany and the German-speaking part of Switzerland. Inclusion criteria were: being employed, working more than 20 h per week, and being within the age range of 18–65 years. Participants were recruited through panel data service provider Respondi (respondi.com). Data were collected via an online questionnaire using web-based survey provider Alchemer (alchemer.com). Data collection occurred in April 2020 (T1), July 2020 (T2) and December 2020 (T3). Participation was voluntary and rewarded with a minimal incentive for completing the survey (i.e. points that could be redeemed toward a given service after completing several surveys). Each participant in the online panel service database had a unique code that ensured anonymity and prevented multiple submissions from one participant. Important items in the survey were mandatory and participants were informed if they accidently skipped an item. Several disqualifying items (e.g. ‘Please choose number three as an answer to this item’) were included as a quality check to exclude participants who give random answers. We calculated that a minimum required sample size for our model with 9 latent variables and 66 parameters is 489 participants (Westland, 2010). Our sample included 2217 participants who completed the questionnaire in the first wave, 1727 completed the questionnaire in second wave and 1196 in third wave (i.e. 47% drop out across the three data collection points). Of the 2217 participants, 81% lived in Germany, 57% were male, 4% completed primary education, 57% secondary education and 39% held a degree from a higher education institute, the mean age was 47.8 years (SD = 10.4).

### Measures

#### Sense of coherence

SOC was assessed using the SOC-L9 scale (Schumacher *et al.*, 2000). This scale has been validated in a large, representative community sample of the German population and comprises those nine items with the highest item-total correlation with Antonovsky’s (Antonovsky, 1987) original 29-item Orientation to Life questionnaire. The short version has a unidimensional structure, but all three dimensions are included in the nine items: comprehensibility (e.g. ‘Do you have the feeling that you are in an unfamiliar situation and don’t know what to do?’; 1 = *very often* to 7 = *very seldom or never*), manageability (e.g. ‘When you think of difficulties you are likely to face in important aspects of your life, do you have the feeling that …’; 1 = *you won’t succeed in overcoming the difficulties* to 7 = *you will always succeed in overcoming the difficulties*) and meaningfulness (e.g. ‘Doing the things you do every day is …’; 1 = *a source of pain and boredom* to 7 = *a source of deep pleasure and satisfaction*).

#### Off-job crafting

The 18-item version of the Needs-Based Off-Job Crafting Scale (Kujanpää *et al.*, 2022) was used to measure OJC over the past month. The items measure the extent to which people proactively seek to satisfy their psychological needs during their non-working time on a Likert-type scale ranging from 1 = *never* to 5 = *very often*. The scale is composed of the six dimensions of the DRAMMA model (Newman *et al.*, 2014) and includes three items per dimension: detachment (e.g. ‘I’ve arranged my off-job time so that I distance myself from work-related tasks’), relaxation (e.g. ‘I’ve planned my off-job activities so that I get relief from stress’), autonomy (e.g. ‘I’ve planned my off-job activities so that I experience control over my life’), mastery (e.g. ‘I’ve arranged my off-job time so that I experience proficiency in the things I undertake’), meaning (e.g. ‘I’ve organized my off-job activities so that I achieve a sense of purpose in what I am doing’) and affiliation (e.g. ‘I’ve made sure to experience close connections to the people around me during off-job time’).

#### Mental well-being

MWB was assessed with the Warwick-Edinburgh Mental Well-Being Scale (WEMWBS) (Tennant *et al.*, 2007). We used the German translation of the seven-item short version of the WEMWBS (Lang and Bachinger, 2017). WEMWBS is a measure of MWB capturing the positive aspects of mental health during the past 2 weeks: positive affect (e.g. ‘I have been feeling optimistic about the future’), satisfying interpersonal relationships (e.g. ‘I have been feeling close to other people’) and positive functioning (e.g. ‘I have been dealing with problems well’). The response scale ranges from 1 = *never* to 5 = *all the time*.

### Statistical and analytical procedure

Data analysis was carried out using R version 4.0.2 (R Core Team, 2020). Specifically, the *cfa* and *sem* functions from the *lavaan* package (Rosseel, 2012) were used to perform confirmatory factor analysis and structural equation modeling using the maximum likelihood estimation procedure. SOC was indicated as a first-order latent variable by the nine items of the scale, OJC was indicated as a second-order latent variable by the mean scores of the six DRAMMA dimensions (three items per dimension) and MWB was indicated as a first-order latent variable by the seven items of the scale.

**Fig. 1:**
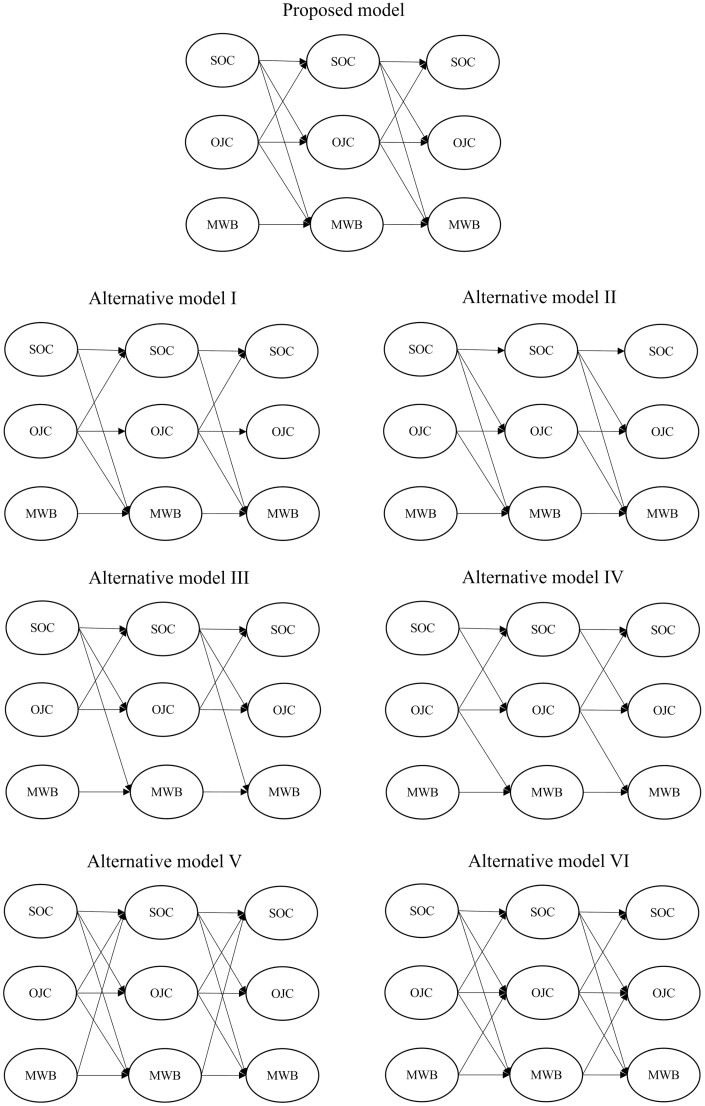
Proposed and alternative structural models.

To test the hypotheses, we compared our proposed model with six alternative models (see Figure 1), where we systematically dropped hypothesized paths (i.e. tested more parsimonious nested models) or included additional paths (i.e. tested more complex nested models). All models were estimated using full information maximum likelihood estimator (FIML). The FIML procedure is strongly recommended in the literature on missing data in longitudinal studies as it has a high parameter and model fit estimation efficiency in the case of time-specific dropouts even in case of up to 50% attrition in sample size (Enders and Bandalos, 2001).

To assess the model fit, we used the comparative-fit index (CFI), root mean square error of approximation (RMSEA) and standardized root mean square residual (SRMR) with the conventional cutoff values. Goodness-of-fit values for CFI surpassing 0.90 indicate an acceptable fit and those exceeding 0.95 indicate a good fit (Bentler and Bonett, 1980). A value under 0.08 for SRMR and RMSEA indicates a good fit (Beauducel and Wittmann, 2005). The models were compared using chi-square difference tests. The error terms of the indicators and of the first-order latent factors were allowed to covary with the corresponding error terms of the other two waves; correlations between the second-order latent factors at T1 and between their error terms at T2 and T3 were also allowed (Russell *et al.*, 1998). Equality constraints were set on the cross-lagged effects between variables from T1 to T2 and from T2 to T3. To test the significance of the indirect effect, we used 5000 bootstrapped samples (Cheung and Lau, 2008) and calculated the indirect effect of SOC on MWB via OJC by multiplying the *β*-values of the single paths from SOC to OJC (T1 → T2) and from OJC to MWB (T2 → T3). A file with an R code for the data analysis is available as a Supplementary Material.

## RESULTS

### Descriptive statistics

Means, standard deviations, Cronbach’s alphas and correlations between the study variables are presented in Table 1. Cronbach’s alphas were good for all variables and for all three measurement points (between 0.84 and 0.91) (Cortina, 1993). All study variables were correlated positively with each other. Test–retest correlations between T1, T2 and T3 variables ranged from 0.66 to 0.81 for SOC, 0.59 to 0.65 for OJC and 0.73 to 0.77 for MWB, indicating high stability.

**Table 1:** Minimum, maximum, means, standard deviations, internal consistencies, and correlations between the study variables

	*Min*	*Max*	*M*	*SD*	*α*	1	2	3	4	5	6	7	8	9
1. SOC T1	1	7	5.00	1.05	0.89	1								
2. OJC T1	1	5	3.71	0.63	0.84	0.37	1							
3. MWB T1	1	5	3.67	0.65	0.88	0.69	0.44	1						
4. SOC T2	1	7	5.03	1.10	0.90	0.80	0.34	0.66	1					
5. OJC T2	1	5	3.55	0.58	0.86	0.40	0.62	0.48	0.47	1				
6. MWB T2	1	5	3.69	0.66	0.90	0.69	0.40	0.76	0.74	0.55	1			
7. SOC T3	1	7	5.02	1.07	0.91	0.76	0.31	0.62	0.78	0.42	0.69	1		
8. OJC T3	1	5	3.58	0.61	0.86	0.42	0.59	0.47	0.43	0.65	0.49	0.50	1	
9. MWB T3	1	5	3.69	0.66	0.90	0.65	0.38	0.72	0.67	0.50	0.76	0.75	0.56	1

*Note. N* = 2217; T = time; all correlations are statistically significant at *p* < 0.001.

### Confirmatory factor analysis

Confirmatory factor analyses were performed separately for each concept of SOC, OJC and MWB using data from the first wave (April 2020). SOC was fitted as a unidimensional construct with nine items loading onto a single latent variable (i.e. overall SOC). A good fit was achieved (*χ*^2^(21) = 163.1, CFI = 0.972, RMSEA = 0.07; SRMR = 0.03) with six pairs of items that were allowed to covary, and the covariances reflected the three subdimensions of SOC. The OJC model was fitted using 18 items loading onto six latent variables representing the subdimensions of OJC. The six subdimensions then loaded onto a single second-order latent variable (i.e. overall OJC). A good fit was achieved (*χ*^2^(127) = 432.2, CFI = 0.966, RMSEA = 0.05; SRMR = 0.04) with two pairs of first-order latent variables that were allowed to covary (detachment—relaxation; meaning—affiliation). Finally, MWB was fitted as a unidimensional construct with seven items loading onto a single latent variable (i.e. overall MWB). A good fit was achieved (*χ*^2^(9) = 109.1, CFI = 0.976, RMSEA = 0.09; SRMR = 0.03) with five pairs of items that were allowed to covary.

### Measurement model and time invariance

Before testing the hypotheses, additional confirmatory factor analyses were performed to test the measurement model and time invariance. In the measurement model (M0), all latent variables were allowed to covary. As shown in Table 2, the model showed a good fit to the data. Additionally, the time invariance tests of the factor loadings showed no significant differences between the unconstrained and constrained models (Δ*χ*^2^ = 34.2, Δdf = 38, *p* = 0.64). Therefore, the relation of the latent variables to the manifest variables was constant across the three measurement points (Cole and Maxwell, 2003).

**Table 2:** Comparisons of the proposed and alternative nested models

Model	Description	*χ* ^2^	*df*	CFI	RMSEA	SRMR	M comparison	Δ*χ*^2^	Δ*df*
M0	Measurement model	7719	1938	0.925	0.037	0.055			
M1	Proposed model	8018	1993	0.922	0.037	0.060			
M2	Alternative model I (− SOC → OJC)	8125	1994	0.921	0.037	0.078	**M1** vs. M2	106.9***	1
M3	Alternative model II (− OJC → SOC)	8020	1994	0.922	0.037	0.061	M1 vs. **M3**	1.7 n.s.	1
M4	Alternative model III (− OJC → MWB)	8032	1994	0.922	0.037	0.062	**M1** vs. M4	13.5***	1
M5	Alternative model IV (− SOC → MWB)	8178	1994	0.920	0.037	0.071	**M1** vs. M5	159.4***	1
M6	Alternative model V (+ MWB → SOC)	7956	1992	0.923	0.037	0.059	M1 vs.** M6**	−62.9***	−1
M7	Alternative model VI (+ MWB → OJC)	8013	1992	0.922	0.037	0.060	M1 vs. **M7**	−6.1*	−1
M8	Final model	7938	1992	0.923	0.037	0.058	M1 vs. **M8**	−80.5***	−1

*Note. N* = 2217; superior models are in bold; values were estimated by a full information maximum likelihood approach in R; *p < 0.05; ***p < 0.001; n.s.: not significant.

### Proposed model and alternative models

Our proposed model revealed good fit indices (*χ*^2^(1993) = 8018, CFI = 0.922, RMSEA = 0.037; SRMR = 0.060). We tested it against four more parsimonious and two more complex nested models (see Figure 1). The fit indices of all tested models are shown in Table 2.

Alternative model I excludes the direct effects of SOC on OJC. The model revealed a significantly worse fit to the data than our proposed model (Δ*χ*^2^ = 106.9, Δdf = 1, *p* < 0.001). This indicates that the path from SOC to OJC is empirically relevant and should be kept in the model supporting Hypothesis 2.

Alternative model II excludes the direct effects of OJC on SOC. The model did not fit the data significantly worse than our proposed model (Δ*χ*^2^ = 1.7, Δdf = 1, *p* = 0.19). This indicates that the path from OJC to SOC is not empirically relevant and should not be kept in the model thus rejecting Hypothesis 3.

Alternative model III excludes the direct effects of OJC on MWB. The model revealed a significantly worse fit to the data than our proposed model (Δ*χ*^2^ = 13.5, Δdf = 1, *p* < 0.001). This indicates that the path from OJC to MWB is empirically relevant and should be kept in the model supporting Hypothesis 4.

Alternative model IV excludes the direct effect of SOC on MWB. The model revealed a significantly worse fit to the data than our proposed model (Δ*χ*^2^ = 159.4, Δdf = 1, *p* < 0.001). This indicates that the path from SOC to MWB is empirically relevant and should be kept in the model supporting Hypothesis 5.

Alternative model V adds a direct effect of MWB on SOC. The model revealed a significantly better fit to the data than our proposed model (Δ*χ*^2^ = −62.9, Δdf = −1, *p* < 0.001). This indicates that the path from MWB to SOC is empirically relevant and should be included in the model.

Alternative model VI adds a direct effect of MWB on OJC. The model revealed a significantly better fit to the data than our proposed model (Δ*χ*^2^ = −6.1, Δdf = −1, *p* < 0.05). This indicates that the path from MWB to OJC is empirically relevant and should be included in the model.

### Final model and indirect effect analysis

**Fig. 2:**
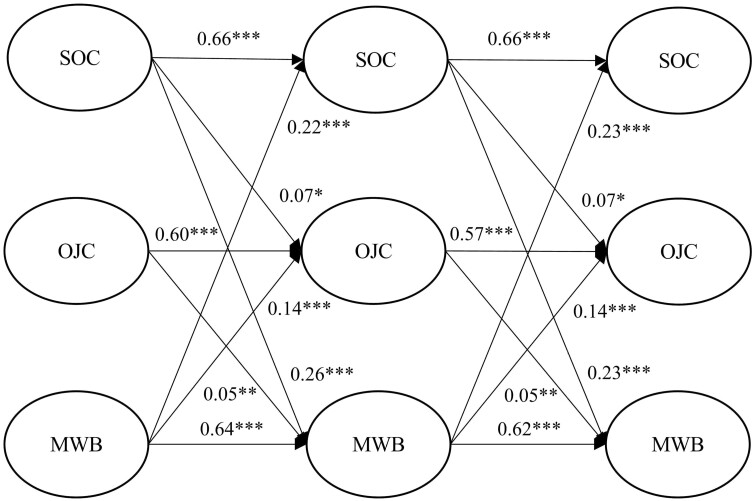
Path coefficients in the final structural model. *Note. N* = 2217; *p < 0.05, **p < 0.01, ***p < 0.001.

The model that fits the data best is our proposed model without the direct path from OJC to SOC and with two additional paths from MWB to SOC and from MWB to OJC (*χ*^2^(1992) = 7938, CFI = 0.923, RMSEA = 0.037; SRMR = 0.058). The path coefficients of the final model are depicted in Figure 2. The autoregressive paths ranged from 0.57 to 0.66 (*p* < 0.001). SOC positively predicted OJC (T1 → T2: *β* = 0.07, *p* < 0.05; T2 → T3: *β* = 0.07, *p* < 0.05) and MWB (T1 → T2: *β* = 0.26, *p* < 0.001; T2 → T3: *β* = 0.23, *p* < 0.001) at all times of measurement. There was also a positive effect of OJC on MWB at all times of measurement (T2: *β* = 0.05, *p* < 0.01; T3: *β* = 0.05, *p* < 0.01). Finally, MWB had a positive effect on OJC (T1 → T2: *β* = 0.14, *p* < 0.001; T2 → T3: *β* = 0.14, *p* < 0.001) and SOC (T1 → T2: *β* = 0.22, *p* < 0.001; T2 → T3: *β* = 0.23, *p* < 0.001) at both times.

The results from bootstrapping showed that the estimated indirect effect from SOC (T1) to MWB (T3) through OJC (T2) was significant (*β* = 0.018, *p* < 0.05), 95% confidence interval [0.003, 0.025], supporting Hypothesis 1.

## DISCUSSION

The purpose of our study was to advance research on the salutogenic model of health by (i) presenting our theoretical assumptions about a path of positive health development using the case of OJC, and (ii) to empirically test these assumptions using a longitudinal three-wave panel study design. Specifically, we examined OJC as a possible underlying mechanism of how SOC translates into positive health outcomes in terms of MWB.

The first aim was achieved by presenting relevant evidence form the fields of salutogenesis and crafting research. We reviewed the development of the salutogenic model of health from the original conception that understands SOC as a protective factor against adversity (Antonovsky, 1979, 1987) to more recent conceptions that propose expanding the model toward positive health development (Bauer *et al.*, 2006, 2019). Additionally, we reviewed the literature from occupational health psychology on crafting and provided arguments for its relevance for salutogenesis. We concluded the literature review by presenting a set of hypotheses about the associations between SOC, OJC and MWB that represent a path of positive health development.

Our findings support our core assumption that OJC partially mediates the relationship between SOC and MWB. In other words, we could show that OJC is one underlying mechanism of how SOC translates into MWB. Individuals with a strong SOC are able to identify their needs and goals and undertake crafting efforts that lead to their needs satisfaction and goal attainment which, in turn, positively affects their MWB. This indicates that SOC functions not only as a protective factor against stress, as was originally conceptualized (Antonovsky, 1979), but also as a personal resource that allows individuals to proactively pursue their goals and needs. In line with the Conservation of Resources Theory (Hobfoll, 2001), those who already possess resources (i.e. a strong SOC) are likely to use (i.e. OJC) and gain new resources in the future. Our findings thus add to the evidence on the positive association between SOC and well-being outcomes (Eriksson and Lindström, 2006; Vogt *et al.*, 2015; Eriksson, 2022), as well as OJC and well-being outcomes (Kujanpää *et al.*, 2020). To our knowledge, our study is the first to explore the association between SOC and crafting (i.e. OJC), and provides evidence that OJC is one underlying mechanism that explains the link between SOC and MWB. It is important to highlight, however, that the indirect effect was relatively small suggesting that OJC explains only a fraction of the association between SOC and MWB.

We did not find support for our third hypothesis that OJC has a direct effect on SOC. Our model thus suggests that the relationship between OJC and SOC is unidirectional, meaning that a strong SOC is predictive of OJC, but OJC does not directly lead to a higher level of SOC. A possible explanation could be that OJC is associated with more immediate outcomes such as needs satisfaction and well-being as has also been shown in previous studies (Kujanpää *et al.*, 2020). However, it may also require longer time periods than our time intervals for a measurable effect of OJC on more stable personal resources such as SOC.

In addition, we found a significant direct effect of MWB on both OJC and SOC suggesting that the relationship between MWB and these variables is reciprocal. High MWB thus contributes to the perception of one’s life as being comprehensible, manageable and meaningful. Both reciprocal relationships are in line with the positive circle in Broaden-and-Build theory (Fredrickson, 2001), as those who experience positive emotions (i.e. MWB) are more likely to broaden their thought and action repertoires (i.e. OJC) and to build up new enduring personal resources (i.e. SOC).

### Limitations, strengths, and directions for future research

The contribution of our study should be considered in light of its limitations. First, psychological constructs such as SOC or MWB are difficult to measure using methods other than self-reporting; however, self-reported data are subject to a common method bias that may influence results (Podsakoff *et al.*, 2003). To minimize the risk, we implemented various strategies in the questionnaire, such as randomization of items, use of disqualifying items (e.g. ‘please choose option number 2’), varying scale length and anchors or reverse-scored items. To check the effectiveness of these procedural remedies, we also empirically tested our data for common method bias using Harman’s single factor approach (Podsakoff *et al.*, 2003), which indicated that common method bias was not an issue in our study (i.e. less than 50% variance explained by a single factor).

Second, the cross-lagged effects we found between the three variables were small. Psychological constructs such as SOC and MWB remain relatively stable over time (Taris and Kompier, 2006) and most of the variance in our studied variables could be explained by their respective baselines. However, we still found significant cross-lagged effects between the variables even when controlling for baselines and with relatively short-time intervals. This indicates that the relationships between SOC, OJC and MWB are relevant.

Third, we collected the data between April and December 2020, during the acute phases of the Covid-19 pandemic. The pandemic could have impacted individuals’ MWB and particularly their crafting behaviors. The restrictive governmental measures imposed to tackle the virus may have significantly restricted individuals’ possibilities for crafting. This may have led to overall lower OJC scores than we would obtain under normal circumstances. However, this would rather lead to an underestimation of the studied relationships.

Finally, even though we used three waves of measurement and a full panel design to test our assumptions, it might still be the case that a third variable (e.g. personality traits) could explain the relationships found. Indeed, as shown in the mediation analysis there is still a large amount of shared variance between SOC and MWB that is not explained by OJC.

Despite the study limitations, the longitudinal study design and the relatively large and heterogeneous sample across the three waves obtained through a professional panel data service are key strengths of our study. We were able to empirically test our hypothesized model and draw meaningful and relevant conclusions of interest not only to salutogenesis researchers but to any researcher studying the working population.

Our study should be replicated with data from a different population and/or with data collected during a post-Covid period. Additionally, future research could examine whether a similar path of positive health development could be identified also in the work domain through job crafting. Research should also study the potential impact of crafting interventions and if individuals with initial low levels of crafting would particularly benefit. It would also be interesting to explore whether data with longer time intervals between waves could identify a positive link between OJC and SOC, which we hypothesized but did not find in our data. Finally, we encourage future researcher to investigate further paths of positive health development in the salutogenic model beyond crafting.

## CONCLUSION

Our study shows that even in a relatively short term, OJC partially mediates the long-observed relationship between SOC and positive individual outcomes such as MWB. This provides evidence for one mechanism underlying the recently postulated path of positive health development in the salutogenic model. For practice, our findings imply establishing ways for supporting people in routinely crafting their non-work domain, to promote their health and well-being. For instance, digital interventions could guide individuals or groups in recognizing and understanding their needs and goals according to the DRAMMA model (Newman *et al.*, 2014) and in identifying need-congruent activities that would lead to their needs satisfaction. This could benefit both their life satisfaction, personal development and overall well-being.

## Supplementary Material

daac159_suppl_Supplementary_Material
