# Supplementary material for: Sense of coherence, off-job crafting, and mental well-being: A path of positive health development
Source: Health Promot Int. 2022 Nov 28;37(6):daac159. doi: 10.1093/heapro/daac159 (PMC9703811; doi:10.1093/heapro/daac159)
Supplement: daac159_suppl_Supplementary_Material [file daac159_suppl_supplementary_material.pdf]

REVISION: Sense of coherence, off-job crafting, and mental well-being: a path of positive health development

Anonymous

26/07/2022

```
suppressMessages(library(tidyverse))
suppressMessages(library(Hmisc))
suppressMessages(library(psych))
suppressMessages(library(lavaan))
suppressMessages(library(readr))
suppressMessages(library(skimr))
suppressWarnings(df <- read_csv("data/wave_456_full.csv"))
```

Cross-lagged panel models **1a) Measurement model**

```
# Model specification
model <- '
          # Defining latent variables
          WB.1 =~ s91_01.4 + s91_02.4 + s91_03.4 + s91_04.4 + s91_05.4 + s91_06.4 + s91_07.4
          WB.2 =~ s91_01.5 + s91_02.5 + s91_03.5 + s91_04.5 + s91_05.5 + s91_06.5 + s91_07.5
          WB.3 =~ s91_01.6 + s91_02.6 + s91_03.6 + s91_04.6 + s91_05.6 + s91_06.6 + s91_07.6
          SoC.1 =~ s51_01.4 + s51_02.4 + s51_03.4 + s51_04.4 + s51_05.4 + s51_06.4 + s51_07.4 + s51_08.4
          SoC.2 =~ s51_01.5 + s51_02.5 + s51_03.5 + s51_04.5 + s51_05.5 + s51_06.5 + s51_07.5 + s51_08.5
          SoC.3 =~ s51_01.6 + s51_02.6 + s51_03.6 + s51_04.6 + s51_05.6 + s51_06.6 + s51_07.6 + s51_08.6
          OJC.1 =~ s200_de.4 + s200_re.4 + s200_au.4 + s200_me.4 + s200_ma.4 + s200_af.4
          OJC.2 =~ s200_de.5 + s200_re.5 + s200_au.5 + s200_me.5 + s200_ma.5 + s200_af.5
          OJC.3 =~ s200_de.6 + s200_re.6 + s200_au.6 + s200_me.6 + s200_ma.6 + s200_af.6

          # Residual covariances
          s91_01.4 ~~ s91_02.4
          s91_02.4 ~~ s91_06.4
          s91_04.4 ~~ s91_05.4
          s91_04.4 ~~ s91_07.4
          s91_05.4 ~~ s91_07.4
          s91_01.5 ~~ s91_02.5
          s91_02.5 ~~ s91_06.5
          s91_04.5 ~~ s91_05.5
          s91_04.5 ~~ s91_07.5
          s91_05.5 ~~ s91_07.5
          s91_01.6 ~~ s91_02.6
          s91_02.6 ~~ s91_06.6
          s91_04.6 ~~ s91_05.6
          s91_04.6 ~~ s91_07.6
          s91_05.6 ~~ s91_07.6
```

```
s91_01.4 ~~ s91_01.5 + s91_01.6
s91_01.5 ~~ s91_01.6
s91_02.4 ~~ s91_02.5 + s91_02.6
s91_02.5 ~~ s91_02.6
s91_03.4 ~~ s91_03.5 + s91_03.6
s91_03.5 ~~ s91_03.6
s91_04.4 ~~ s91_04.5 + s91_04.6
s91_04.5 ~~ s91_04.6
s91_05.4 ~~ s91_05.5 + s91_05.6
s91_05.5 ~~ s91_05.6
s91_06.4 ~~ s91_06.5 + s91_06.6
s91_06.5 ~~ s91_06.6
s91_07.4 ~~ s91_07.5 + s91_07.6
s91_07.5 ~~ s91_07.6
s200_de.4 ~~ s200_re.4
s200_de.5 ~~ s200_re.5
s200_de.6 ~~ s200_re.6
s200_me.4 ~~ s200_af.4
s200_me.5 ~~ s200_af.5
s200_me.6 ~~ s200_af.6
s200_de.4 ~~ s200_de.5 + s200_de.6
s200_de.5 ~~ s200_de.6
s200_re.4 ~~ s200_re.5 + s200_re.6
s200_re.5 ~~ s200_re.6
s200_au.4 ~~ s200_au.5 + s200_au.6
s200_au.5 ~~ s200_au.6
s200_me.4 ~~ s200_me.5 + s200_me.6
s200_me.5 ~~ s200_me.6
s200_ma.4 ~~ s200_ma.5 + s200_ma.6
s200_ma.5 ~~ s200_ma.6
s200_af.4 ~~ s200_af.5 + s200_af.6
s200_af.5 ~~ s200_af.6
s51_01.4 ~~ s51_04.4
s51_01.4 ~~ s51_07.4
s51_01.4 ~~ s51_09.4
s51_04.4 ~~ s51_07.4
s51_04.4 ~~ s51_09.4
s51_07.4 ~~ s51_09.4
s51_01.5 ~~ s51_04.5
s51_01.5 ~~ s51_07.5
s51_01.5 ~~ s51_09.5
s51_04.5 ~~ s51_07.5
s51_04.5 ~~ s51_09.5
s51_07.5 ~~ s51_09.5
s51_01.6 ~~ s51_04.6
s51_01.6 ~~ s51_07.6
s51_01.6 ~~ s51_09.6
s51_04.6 ~~ s51_07.6
s51_04.6 ~~ s51_09.6
s51_07.6 ~~ s51_09.6
s51_01.4 ~~ s51_01.5 + s51_01.6
s51_01.5 ~~ s51_01.6
s51_02.4 ~~ s51_02.5 + s51_02.6
```

```
          s51_02.5 ~~ s51_02.6
          s51_03.4 ~~ s51_03.5 + s51_03.6
          s51_03.5 ~~ s51_03.6
          s51_04.4 ~~ s51_04.5 + s51_04.6
          s51_04.5 ~~ s51_04.6
          s51_05.4 ~~ s51_05.5 + s51_05.6
          s51_05.5 ~~ s51_05.6
          s51_06.4 ~~ s51_06.5 + s51_06.6
          s51_06.5 ~~ s51_06.6
          s51_07.4 ~~ s51_07.5 + s51_07.6
          s51_07.5 ~~ s51_07.6
          s51_08.4 ~~ s51_08.5 + s51_08.6
          s51_08.5 ~~ s51_08.6
          s51_09.4 ~~ s51_09.5 + s51_09.6
          s51_09.5 ~~ s51_09.6
'

#  Model estimation
fit0a <- cfa(model,
             data = df,
             estimator = "ML",
             missing = "ML")

summary(fit0a,
        rsquare = TRUE,
        standardized = TRUE,
        fit.measures = TRUE)
```

1b) Measurement model with fixed loadings across the three time points

```
# Model specification
model <- '
          # Defining latent variables
          WB.1 =~ a1*s91_01.4 + a2*s91_02.4 + a3*s91_03.4 + a4*s91_04.4 + a5*s91_05.4 + a6*s91_06.4 + a
          WB.2 =~ a1*s91_01.5 + a2*s91_02.5 + a3*s91_03.5 + a4*s91_04.5 + a5*s91_05.5 + a6*s91_06.5 + a
          WB.3 =~ a1*s91_01.6 + a2*s91_02.6 + a3*s91_03.6 + a4*s91_04.6 + a5*s91_05.6 + a6*s91_06.6 + a
          SoC.1 =~ b1*s51_01.4 + b2*s51_02.4 + b3*s51_03.4 + b4*s51_04.4 + b5*s51_05.4 + b6*s51_06.4 +
          SoC.2 =~ b1*s51_01.5 + b2*s51_02.5 + b3*s51_03.5 + b4*s51_04.5 + b5*s51_05.5 + b6*s51_06.5 +
          SoC.3 =~ b1*s51_01.6 + b2*s51_02.6 + b3*s51_03.6 + b4*s51_04.6 + b5*s51_05.6 + b6*s51_06.6 +
          OJC.1 =~ c1*s200_de.4 + c2*s200_re.4 + c3*s200_au.4 + c4*s200_me.4 + c5*s200_ma.4 + c6*s200_a
          OJC.2 =~ c1*s200_de.5 + c2*s200_re.5 + c3*s200_au.5 + c4*s200_me.5 + c5*s200_ma.5 + c6*s200_a
          OJC.3 =~ c1*s200_de.6 + c2*s200_re.6 + c3*s200_au.6 + c4*s200_me.6 + c5*s200_ma.6 + c6*s200_a

          # Residual covariances
          s91_01.4 ~~ s91_02.4
          s91_02.4 ~~ s91_06.4
          s91_04.4 ~~ s91_05.4
          s91_04.4 ~~ s91_07.4
          s91_05.4 ~~ s91_07.4
          s91_01.5 ~~ s91_02.5
          s91_02.5 ~~ s91_06.5
          s91_04.5 ~~ s91_05.5
          s91_04.5 ~~ s91_07.5
          s91_05.5 ~~ s91_07.5
```

```
s91_01.6 ~~ s91_02.6
s91_02.6 ~~ s91_06.6
s91_04.6 ~~ s91_05.6
s91_04.6 ~~ s91_07.6
s91_05.6 ~~ s91_07.6
s91_01.4 ~~ s91_01.5 + s91_01.6
s91_01.5 ~~ s91_01.6
s91_02.4 ~~ s91_02.5 + s91_02.6
s91_02.5 ~~ s91_02.6
s91_03.4 ~~ s91_03.5 + s91_03.6
s91_03.5 ~~ s91_03.6
s91_04.4 ~~ s91_04.5 + s91_04.6
s91_04.5 ~~ s91_04.6
s91_05.4 ~~ s91_05.5 + s91_05.6
s91_05.5 ~~ s91_05.6
s91_06.4 ~~ s91_06.5 + s91_06.6
s91_06.5 ~~ s91_06.6
s91_07.4 ~~ s91_07.5 + s91_07.6
s91_07.5 ~~ s91_07.6
s200_de.4 ~~ s200_re.4
s200_de.5 ~~ s200_re.5
s200_de.6 ~~ s200_re.6
s200_me.4 ~~ s200_af.4
s200_me.5 ~~ s200_af.5
s200_me.6 ~~ s200_af.6
s200_de.4 ~~ s200_de.5 + s200_de.6
s200_de.5 ~~ s200_de.6
s200_re.4 ~~ s200_re.5 + s200_re.6
s200_re.5 ~~ s200_re.6
s200_au.4 ~~ s200_au.5 + s200_au.6
s200_au.5 ~~ s200_au.6
s200_me.4 ~~ s200_me.5 + s200_me.6
s200_me.5 ~~ s200_me.6
s200_ma.4 ~~ s200_ma.5 + s200_ma.6
s200_ma.5 ~~ s200_ma.6
s200_af.4 ~~ s200_af.5 + s200_af.6
s200_af.5 ~~ s200_af.6
s51_01.4 ~~ s51_04.4
s51_01.4 ~~ s51_07.4
s51_01.4 ~~ s51_09.4
s51_04.4 ~~ s51_07.4
s51_04.4 ~~ s51_09.4
s51_07.4 ~~ s51_09.4
s51_01.5 ~~ s51_04.5
s51_01.5 ~~ s51_07.5
s51_01.5 ~~ s51_09.5
s51_04.5 ~~ s51_07.5
s51_04.5 ~~ s51_09.5
s51_07.5 ~~ s51_09.5
s51_01.6 ~~ s51_04.6
s51_01.6 ~~ s51_07.6
s51_01.6 ~~ s51_09.6
s51_04.6 ~~ s51_07.6
```

```
          s51_04.6 ~~ s51_09.6
          s51_07.6 ~~ s51_09.6
          s51_01.4 ~~ s51_01.5 + s51_01.6
          s51_01.5 ~~ s51_01.6
          s51_02.4 ~~ s51_02.5 + s51_02.6
          s51_02.5 ~~ s51_02.6
          s51_03.4 ~~ s51_03.5 + s51_03.6
          s51_03.5 ~~ s51_03.6
          s51_04.4 ~~ s51_04.5 + s51_04.6
          s51_04.5 ~~ s51_04.6
          s51_05.4 ~~ s51_05.5 + s51_05.6
          s51_05.5 ~~ s51_05.6
          s51_06.4 ~~ s51_06.5 + s51_06.6
          s51_06.5 ~~ s51_06.6
          s51_07.4 ~~ s51_07.5 + s51_07.6
          s51_07.5 ~~ s51_07.6
          s51_08.4 ~~ s51_08.5 + s51_08.6
          s51_08.5 ~~ s51_08.6
          s51_09.4 ~~ s51_09.5 + s51_09.6
          s51_09.5 ~~ s51_09.6
'

#  Model estimation
fit0b <- cfa(model,
             data = df,
             estimator = "ML",
             missing = "ML")

summary(fit0b,
        rsquare = TRUE,
        standardized = TRUE,
        fit.measures = TRUE)
```

2) Proposed model (M1)

```
# Model specification
model1 <- '
          # Defining latent variables
          WB.1 =~ a1*s91_01.4 + a2*s91_02.4 + a3*s91_03.4 + a4*s91_04.4 + a5*s91_05.4 + a6*s91_06.4 + a
          WB.2 =~ a1*s91_01.5 + a2*s91_02.5 + a3*s91_03.5 + a4*s91_04.5 + a5*s91_05.5 + a6*s91_06.5 + a
          WB.3 =~ a1*s91_01.6 + a2*s91_02.6 + a3*s91_03.6 + a4*s91_04.6 + a5*s91_05.6 + a6*s91_06.6 + a
          SoC.1 =~ b1*s51_01.4 + b2*s51_02.4 + b3*s51_03.4 + b4*s51_04.4 + b5*s51_05.4 + b6*s51_06.4 +
          SoC.2 =~ b1*s51_01.5 + b2*s51_02.5 + b3*s51_03.5 + b4*s51_04.5 + b5*s51_05.5 + b6*s51_06.5 +
          SoC.3 =~ b1*s51_01.6 + b2*s51_02.6 + b3*s51_03.6 + b4*s51_04.6 + b5*s51_05.6 + b6*s51_06.6 +
          OJC.1 =~ c1*s200_de.4 + c2*s200_re.4 + c3*s200_au.4 + c4*s200_me.4 + c5*s200_ma.4 + c6*s200_a
          OJC.2 =~ c1*s200_de.5 + c2*s200_re.5 + c3*s200_au.5 + c4*s200_me.5 + c5*s200_ma.5 + c6*s200_a
          OJC.3 =~ c1*s200_de.6 + c2*s200_re.6 + c3*s200_au.6 + c4*s200_me.6 + c5*s200_ma.6 + c6*s200_a

          # Structural model
          SoC.2 ~ SoC.1 + a*OJC.1
          SoC.3 ~ SoC.2 + a*OJC.2
          OJC.2 ~ OJC.1 + b*SoC.1
          OJC.3 ~ OJC.2 + b*SoC.2
          WB.2 ~ WB.1 + c*OJC.1 + d*SoC.1
```

```
WB.3 ~ WB.2 + c*OJC.2 + d*SoC.2

# Covariances
SoC.1 ~~ OJC.1 + WB.1
OJC.1 ~~ WB.1
SoC.2 ~~ OJC.2 + WB.2
OJC.2 ~~ WB.2
SoC.3 ~~ OJC.3 + WB.3
OJC.3 ~~ WB.3

# Residual covariances
s91_01.4 ~~ s91_02.4
s91_02.4 ~~ s91_06.4
s91_04.4 ~~ s91_05.4
s91_04.4 ~~ s91_07.4
s91_05.4 ~~ s91_07.4
s91_01.5 ~~ s91_02.5
s91_02.5 ~~ s91_06.5
s91_04.5 ~~ s91_05.5
s91_04.5 ~~ s91_07.5
s91_05.5 ~~ s91_07.5
s91_01.6 ~~ s91_02.6
s91_02.6 ~~ s91_06.6
s91_04.6 ~~ s91_05.6
s91_04.6 ~~ s91_07.6
s91_05.6 ~~ s91_07.6
s91_01.4 ~~ s91_01.5 + s91_01.6
s91_01.5 ~~ s91_01.6
s91_02.4 ~~ s91_02.5 + s91_02.6
s91_02.5 ~~ s91_02.6
s91_03.4 ~~ s91_03.5 + s91_03.6
s91_03.5 ~~ s91_03.6
s91_04.4 ~~ s91_04.5 + s91_04.6
s91_04.5 ~~ s91_04.6
s91_05.4 ~~ s91_05.5 + s91_05.6
s91_05.5 ~~ s91_05.6
s91_06.4 ~~ s91_06.5 + s91_06.6
s91_06.5 ~~ s91_06.6
s91_07.4 ~~ s91_07.5 + s91_07.6
s91_07.5 ~~ s91_07.6
s200_de.4 ~~ s200_re.4
s200_de.5 ~~ s200_re.5
s200_de.6 ~~ s200_re.6
s200_me.4 ~~ s200_af.4
s200_me.5 ~~ s200_af.5
s200_me.6 ~~ s200_af.6
s200_de.4 ~~ s200_de.5 + s200_de.6
s200_de.5 ~~ s200_de.6
s200_re.4 ~~ s200_re.5 + s200_re.6
s200_re.5 ~~ s200_re.6
s200_au.4 ~~ s200_au.5 + s200_au.6
s200_au.5 ~~ s200_au.6
s200_me.4 ~~ s200_me.5 + s200_me.6
```

```
          s200_me.5 ~~ s200_me.6
          s200_ma.4 ~~ s200_ma.5 + s200_ma.6
          s200_ma.5 ~~ s200_ma.6
          s200_af.4 ~~ s200_af.5 + s200_af.6
          s200_af.5 ~~ s200_af.6
          s51_01.4 ~~ s51_04.4
          s51_01.4 ~~ s51_07.4
          s51_01.4 ~~ s51_09.4
          s51_04.4 ~~ s51_07.4
          s51_04.4 ~~ s51_09.4
          s51_07.4 ~~ s51_09.4
          s51_01.5 ~~ s51_04.5
          s51_01.5 ~~ s51_07.5
          s51_01.5 ~~ s51_09.5
          s51_04.5 ~~ s51_07.5
          s51_04.5 ~~ s51_09.5
          s51_07.5 ~~ s51_09.5
          s51_01.6 ~~ s51_04.6
          s51_01.6 ~~ s51_07.6
          s51_01.6 ~~ s51_09.6
          s51_04.6 ~~ s51_07.6
          s51_04.6 ~~ s51_09.6
          s51_07.6 ~~ s51_09.6
          s51_01.4 ~~ s51_01.5 + s51_01.6
          s51_01.5 ~~ s51_01.6
          s51_02.4 ~~ s51_02.5 + s51_02.6
          s51_02.5 ~~ s51_02.6
          s51_03.4 ~~ s51_03.5 + s51_03.6
          s51_03.5 ~~ s51_03.6
          s51_04.4 ~~ s51_04.5 + s51_04.6
          s51_04.5 ~~ s51_04.6
          s51_05.4 ~~ s51_05.5 + s51_05.6
          s51_05.5 ~~ s51_05.6
          s51_06.4 ~~ s51_06.5 + s51_06.6
          s51_06.5 ~~ s51_06.6
          s51_07.4 ~~ s51_07.5 + s51_07.6
          s51_07.5 ~~ s51_07.6
          s51_08.4 ~~ s51_08.5 + s51_08.6
          s51_08.5 ~~ s51_08.6
          s51_09.4 ~~ s51_09.5 + s51_09.6
          s51_09.5 ~~ s51_09.6
'

#  Model estimation
fit1 <- sem(model1,
            data = df,
            estimator = "ML",
            missing = "ML")

summary(fit1,
        rsquare = TRUE,
        standardized = TRUE,
        fit.measures = TRUE)
```

3) Alternative model I: Without path SOC -> OJC

```
# Model specification
model1 <- '
          # Defining latent variables
          WB.1 =~ a1*s91_01.4 + a2*s91_02.4 + a3*s91_03.4 + a4*s91_04.4 + a5*s91_05.4 + a6*s91_06.4 + a
          WB.2 =~ a1*s91_01.5 + a2*s91_02.5 + a3*s91_03.5 + a4*s91_04.5 + a5*s91_05.5 + a6*s91_06.5 + a
          WB.3 =~ a1*s91_01.6 + a2*s91_02.6 + a3*s91_03.6 + a4*s91_04.6 + a5*s91_05.6 + a6*s91_06.6 + a
          SoC.1 =~ b1*s51_01.4 + b2*s51_02.4 + b3*s51_03.4 + b4*s51_04.4 + b5*s51_05.4 + b6*s51_06.4 +
          SoC.2 =~ b1*s51_01.5 + b2*s51_02.5 + b3*s51_03.5 + b4*s51_04.5 + b5*s51_05.5 + b6*s51_06.5 +
          SoC.3 =~ b1*s51_01.6 + b2*s51_02.6 + b3*s51_03.6 + b4*s51_04.6 + b5*s51_05.6 + b6*s51_06.6 +
          OJC.1 =~ c1*s200_de.4 + c2*s200_re.4 + c3*s200_au.4 + c4*s200_me.4 + c5*s200_ma.4 + c6*s200_a
          OJC.2 =~ c1*s200_de.5 + c2*s200_re.5 + c3*s200_au.5 + c4*s200_me.5 + c5*s200_ma.5 + c6*s200_a
          OJC.3 =~ c1*s200_de.6 + c2*s200_re.6 + c3*s200_au.6 + c4*s200_me.6 + c5*s200_ma.6 + c6*s200_a

          # Structural model
          SoC.2 ~ SoC.1 + a*OJC.1
          SoC.3 ~ SoC.2 + a*OJC.2
          OJC.2 ~ OJC.1
          OJC.3 ~ OJC.2
          WB.2 ~ WB.1 + c*OJC.1 + d*SoC.1
          WB.3 ~ WB.2 + c*OJC.2 + d*SoC.2

          # Covariances
          SoC.1 ~~ OJC.1 + WB.1
          OJC.1 ~~ WB.1
          SoC.2 ~~ OJC.2 + WB.2
          OJC.2 ~~ WB.2
          SoC.3 ~~ OJC.3 + WB.3
          OJC.3 ~~ WB.3

          # Residual covariances
          s91_01.4 ~~ s91_02.4
          s91_02.4 ~~ s91_06.4
          s91_04.4 ~~ s91_05.4
          s91_04.4 ~~ s91_07.4
          s91_05.4 ~~ s91_07.4
          s91_01.5 ~~ s91_02.5
          s91_02.5 ~~ s91_06.5
          s91_04.5 ~~ s91_05.5
          s91_04.5 ~~ s91_07.5
          s91_05.5 ~~ s91_07.5
          s91_01.6 ~~ s91_02.6
          s91_02.6 ~~ s91_06.6
          s91_04.6 ~~ s91_05.6
          s91_04.6 ~~ s91_07.6
          s91_05.6 ~~ s91_07.6
          s91_01.4 ~~ s91_01.5 + s91_01.6
          s91_01.5 ~~ s91_01.6
          s91_02.4 ~~ s91_02.5 + s91_02.6
          s91_02.5 ~~ s91_02.6
          s91_03.4 ~~ s91_03.5 + s91_03.6
          s91_03.5 ~~ s91_03.6
          s91_04.4 ~~ s91_04.5 + s91_04.6
```

```
s91_04.5 ~~ s91_04.6
s91_05.4 ~~ s91_05.5 + s91_05.6
s91_05.5 ~~ s91_05.6
s91_06.4 ~~ s91_06.5 + s91_06.6
s91_06.5 ~~ s91_06.6
s91_07.4 ~~ s91_07.5 + s91_07.6
s91_07.5 ~~ s91_07.6
s200_de.4 ~~ s200_re.4
s200_de.5 ~~ s200_re.5
s200_de.6 ~~ s200_re.6
s200_me.4 ~~ s200_af.4
s200_me.5 ~~ s200_af.5
s200_me.6 ~~ s200_af.6
s200_de.4 ~~ s200_de.5 + s200_de.6
s200_de.5 ~~ s200_de.6
s200_re.4 ~~ s200_re.5 + s200_re.6
s200_re.5 ~~ s200_re.6
s200_au.4 ~~ s200_au.5 + s200_au.6
s200_au.5 ~~ s200_au.6
s200_me.4 ~~ s200_me.5 + s200_me.6
s200_me.5 ~~ s200_me.6
s200_ma.4 ~~ s200_ma.5 + s200_ma.6
s200_ma.5 ~~ s200_ma.6
s200_af.4 ~~ s200_af.5 + s200_af.6
s200_af.5 ~~ s200_af.6
s51_01.4 ~~ s51_04.4
s51_01.4 ~~ s51_07.4
s51_01.4 ~~ s51_09.4
s51_04.4 ~~ s51_07.4
s51_04.4 ~~ s51_09.4
s51_07.4 ~~ s51_09.4
s51_01.5 ~~ s51_04.5
s51_01.5 ~~ s51_07.5
s51_01.5 ~~ s51_09.5
s51_04.5 ~~ s51_07.5
s51_04.5 ~~ s51_09.5
s51_07.5 ~~ s51_09.5
s51_01.6 ~~ s51_04.6
s51_01.6 ~~ s51_07.6
s51_01.6 ~~ s51_09.6
s51_04.6 ~~ s51_07.6
s51_04.6 ~~ s51_09.6
s51_07.6 ~~ s51_09.6
s51_01.4 ~~ s51_01.5 + s51_01.6
s51_01.5 ~~ s51_01.6
s51_02.4 ~~ s51_02.5 + s51_02.6
s51_02.5 ~~ s51_02.6
s51_03.4 ~~ s51_03.5 + s51_03.6
s51_03.5 ~~ s51_03.6
s51_04.4 ~~ s51_04.5 + s51_04.6
s51_04.5 ~~ s51_04.6
s51_05.4 ~~ s51_05.5 + s51_05.6
s51_05.5 ~~ s51_05.6
```

```
          s51_06.4 ~~ s51_06.5 + s51_06.6
          s51_06.5 ~~ s51_06.6
          s51_07.4 ~~ s51_07.5 + s51_07.6
          s51_07.5 ~~ s51_07.6
          s51_08.4 ~~ s51_08.5 + s51_08.6
          s51_08.5 ~~ s51_08.6
          s51_09.4 ~~ s51_09.5 + s51_09.6
          s51_09.5 ~~ s51_09.6
'

#  Model estimation
fit2 <- sem(model1,
            data = df,
            estimator = "ML",
            missing = "ML")

summary(fit2,
        rsquare = TRUE,
        standardized = TRUE,
        fit.measures = TRUE)
```

4) Alternative model II: Without path OJC -> SOC

```
# Model specification
model1 <- '
          # Defining latent variables
          WB.1 =~ a1*s91_01.4 + a2*s91_02.4 + a3*s91_03.4 + a4*s91_04.4 + a5*s91_05.4 + a6*s91_06.4 + a
          WB.2 =~ a1*s91_01.5 + a2*s91_02.5 + a3*s91_03.5 + a4*s91_04.5 + a5*s91_05.5 + a6*s91_06.5 + a
          WB.3 =~ a1*s91_01.6 + a2*s91_02.6 + a3*s91_03.6 + a4*s91_04.6 + a5*s91_05.6 + a6*s91_06.6 + a
          SoC.1 =~ b1*s51_01.4 + b2*s51_02.4 + b3*s51_03.4 + b4*s51_04.4 + b5*s51_05.4 + b6*s51_06.4 + 
          SoC.2 =~ b1*s51_01.5 + b2*s51_02.5 + b3*s51_03.5 + b4*s51_04.5 + b5*s51_05.5 + b6*s51_06.5 + 
          SoC.3 =~ b1*s51_01.6 + b2*s51_02.6 + b3*s51_03.6 + b4*s51_04.6 + b5*s51_05.6 + b6*s51_06.6 + 
          OJC.1 =~ c1*s200_de.4 + c2*s200_re.4 + c3*s200_au.4 + c4*s200_me.4 + c5*s200_ma.4 + c6*s200_a
          OJC.2 =~ c1*s200_de.5 + c2*s200_re.5 + c3*s200_au.5 + c4*s200_me.5 + c5*s200_ma.5 + c6*s200_a
          OJC.3 =~ c1*s200_de.6 + c2*s200_re.6 + c3*s200_au.6 + c4*s200_me.6 + c5*s200_ma.6 + c6*s200_a

          # Structural model
          SoC.2 ~ SoC.1
          SoC.3 ~ SoC.2
          OJC.2 ~ OJC.1 + b*SoC.1
          OJC.3 ~ OJC.2 + b*SoC.2
          WB.2 ~ WB.1 + c*OJC.1 + d*SoC.1
          WB.3 ~ WB.2 + c*OJC.2 + d*SoC.2

          # Covariances
          SoC.1 ~~ OJC.1 + WB.1
          OJC.1 ~~ WB.1
          SoC.2 ~~ OJC.2 + WB.2
          OJC.2 ~~ WB.2
          SoC.3 ~~ OJC.3 + WB.3
          OJC.3 ~~ WB.3

          # Residual covariances
          s91_01.4 ~~ s91_02.4
```

```
s91_02.4 ~~ s91_06.4
s91_04.4 ~~ s91_05.4
s91_04.4 ~~ s91_07.4
s91_05.4 ~~ s91_07.4
s91_01.5 ~~ s91_02.5
s91_02.5 ~~ s91_06.5
s91_04.5 ~~ s91_05.5
s91_04.5 ~~ s91_07.5
s91_05.5 ~~ s91_07.5
s91_01.6 ~~ s91_02.6
s91_02.6 ~~ s91_06.6
s91_04.6 ~~ s91_05.6
s91_04.6 ~~ s91_07.6
s91_05.6 ~~ s91_07.6
s91_01.4 ~~ s91_01.5 + s91_01.6
s91_01.5 ~~ s91_01.6
s91_02.4 ~~ s91_02.5 + s91_02.6
s91_02.5 ~~ s91_02.6
s91_03.4 ~~ s91_03.5 + s91_03.6
s91_03.5 ~~ s91_03.6
s91_04.4 ~~ s91_04.5 + s91_04.6
s91_04.5 ~~ s91_04.6
s91_05.4 ~~ s91_05.5 + s91_05.6
s91_05.5 ~~ s91_05.6
s91_06.4 ~~ s91_06.5 + s91_06.6
s91_06.5 ~~ s91_06.6
s91_07.4 ~~ s91_07.5 + s91_07.6
s91_07.5 ~~ s91_07.6
s200_de.4 ~~ s200_re.4
s200_de.5 ~~ s200_re.5
s200_de.6 ~~ s200_re.6
s200_me.4 ~~ s200_af.4
s200_me.5 ~~ s200_af.5
s200_me.6 ~~ s200_af.6
s200_de.4 ~~ s200_de.5 + s200_de.6
s200_de.5 ~~ s200_de.6
s200_re.4 ~~ s200_re.5 + s200_re.6
s200_re.5 ~~ s200_re.6
s200_au.4 ~~ s200_au.5 + s200_au.6
s200_au.5 ~~ s200_au.6
s200_me.4 ~~ s200_me.5 + s200_me.6
s200_me.5 ~~ s200_me.6
s200_ma.4 ~~ s200_ma.5 + s200_ma.6
s200_ma.5 ~~ s200_ma.6
s200_af.4 ~~ s200_af.5 + s200_af.6
s200_af.5 ~~ s200_af.6
s51_01.4 ~~ s51_04.4
s51_01.4 ~~ s51_07.4
s51_01.4 ~~ s51_09.4
s51_04.4 ~~ s51_07.4
s51_04.4 ~~ s51_09.4
s51_07.4 ~~ s51_09.4
s51_01.5 ~~ s51_04.5
```

```
          s51_01.5 ~~ s51_07.5
          s51_01.5 ~~ s51_09.5
          s51_04.5 ~~ s51_07.5
          s51_04.5 ~~ s51_09.5
          s51_07.5 ~~ s51_09.5
          s51_01.6 ~~ s51_04.6
          s51_01.6 ~~ s51_07.6
          s51_01.6 ~~ s51_09.6
          s51_04.6 ~~ s51_07.6
          s51_04.6 ~~ s51_09.6
          s51_07.6 ~~ s51_09.6
          s51_01.4 ~~ s51_01.5 + s51_01.6
          s51_01.5 ~~ s51_01.6
          s51_02.4 ~~ s51_02.5 + s51_02.6
          s51_02.5 ~~ s51_02.6
          s51_03.4 ~~ s51_03.5 + s51_03.6
          s51_03.5 ~~ s51_03.6
          s51_04.4 ~~ s51_04.5 + s51_04.6
          s51_04.5 ~~ s51_04.6
          s51_05.4 ~~ s51_05.5 + s51_05.6
          s51_05.5 ~~ s51_05.6
          s51_06.4 ~~ s51_06.5 + s51_06.6
          s51_06.5 ~~ s51_06.6
          s51_07.4 ~~ s51_07.5 + s51_07.6
          s51_07.5 ~~ s51_07.6
          s51_08.4 ~~ s51_08.5 + s51_08.6
          s51_08.5 ~~ s51_08.6
          s51_09.4 ~~ s51_09.5 + s51_09.6
          s51_09.5 ~~ s51_09.6
'

#  Model estimation
fit3 <- sem(model1,
            data = df,
            estimator = "ML",
            missing = "ML")

summary(fit3,
        rsquare = TRUE,
        standardized = TRUE,
        fit.measures = TRUE)
```

Alternative model III: Without path OJC -> MWB

```
model1 <- '
          # Defining latent variables
          WB.1 =~ a1*s91_01.4 + a2*s91_02.4 + a3*s91_03.4 + a4*s91_04.4 + a5*s91_05.4 + a6*s91_06.4 + a
          WB.2 =~ a1*s91_01.5 + a2*s91_02.5 + a3*s91_03.5 + a4*s91_04.5 + a5*s91_05.5 + a6*s91_06.5 + a
          WB.3 =~ a1*s91_01.6 + a2*s91_02.6 + a3*s91_03.6 + a4*s91_04.6 + a5*s91_05.6 + a6*s91_06.6 + a
          SoC.1 =~ b1*s51_01.4 + b2*s51_02.4 + b3*s51_03.4 + b4*s51_04.4 + b5*s51_05.4 + b6*s51_06.4 +
          SoC.2 =~ b1*s51_01.5 + b2*s51_02.5 + b3*s51_03.5 + b4*s51_04.5 + b5*s51_05.5 + b6*s51_06.5 +
          SoC.3 =~ b1*s51_01.6 + b2*s51_02.6 + b3*s51_03.6 + b4*s51_04.6 + b5*s51_05.6 + b6*s51_06.6 +
          OJC.1 =~ c1*s200_de.4 + c2*s200_re.4 + c3*s200_au.4 + c4*s200_me.4 + c5*s200_ma.4 + c6*s200_a
```

```
OJC.2 =~ c1*s200_de.5 + c2*s200_re.5 + c3*s200_au.5 + c4*s200_me.5 + c5*s200_ma.5 + c6*s200_a[illegible]
OJC.3 =~ c1*s200_de.6 + c2*s200_re.6 + c3*s200_au.6 + c4*s200_me.6 + c5*s200_ma.6 + c6*s200_a[illegible]

# Structural model
SoC.2 ~ SoC.1 + a*OJC.1
SoC.3 ~ SoC.2 + a*OJC.2
OJC.2 ~ OJC.1 + b*SoC.1
OJC.3 ~ OJC.2 + b*SoC.2
WB.2 ~ WB.1 + c*SoC.1
WB.3 ~ WB.2 + c*SoC.2

# Covariances
SoC.1 ~~ OJC.1 + WB.1
OJC.1 ~~ WB.1
SoC.2 ~~ OJC.2 + WB.2
OJC.2 ~~ WB.2
SoC.3 ~~ OJC.3 + WB.3
OJC.3 ~~ WB.3

# Residual covariances
s91_01.4 ~~ s91_02.4
s91_02.4 ~~ s91_06.4
s91_04.4 ~~ s91_05.4
s91_04.4 ~~ s91_07.4
s91_05.4 ~~ s91_07.4
s91_01.5 ~~ s91_02.5
s91_02.5 ~~ s91_06.5
s91_04.5 ~~ s91_05.5
s91_04.5 ~~ s91_07.5
s91_05.5 ~~ s91_07.5
s91_01.6 ~~ s91_02.6
s91_02.6 ~~ s91_06.6
s91_04.6 ~~ s91_05.6
s91_04.6 ~~ s91_07.6
s91_05.6 ~~ s91_07.6
s91_01.4 ~~ s91_01.5 + s91_01.6
s91_01.5 ~~ s91_01.6
s91_02.4 ~~ s91_02.5 + s91_02.6
s91_02.5 ~~ s91_02.6
s91_03.4 ~~ s91_03.5 + s91_03.6
s91_03.5 ~~ s91_03.6
s91_04.4 ~~ s91_04.5 + s91_04.6
s91_04.5 ~~ s91_04.6
s91_05.4 ~~ s91_05.5 + s91_05.6
s91_05.5 ~~ s91_05.6
s91_06.4 ~~ s91_06.5 + s91_06.6
s91_06.5 ~~ s91_06.6
s91_07.4 ~~ s91_07.5 + s91_07.6
s91_07.5 ~~ s91_07.6
s200_de.4 ~~ s200_re.4
s200_de.5 ~~ s200_re.5
s200_de.6 ~~ s200_re.6
s200_me.4 ~~ s200_af.4
```

```
          s200_me.5 ~~ s200_af.5
          s200_me.6 ~~ s200_af.6
          s200_de.4 ~~ s200_de.5 + s200_de.6
          s200_de.5 ~~ s200_de.6
          s200_re.4 ~~ s200_re.5 + s200_re.6
          s200_re.5 ~~ s200_re.6
          s200_au.4 ~~ s200_au.5 + s200_au.6
          s200_au.5 ~~ s200_au.6
          s200_me.4 ~~ s200_me.5 + s200_me.6
          s200_me.5 ~~ s200_me.6
          s200_ma.4 ~~ s200_ma.5 + s200_ma.6
          s200_ma.5 ~~ s200_ma.6
          s200_af.4 ~~ s200_af.5 + s200_af.6
          s200_af.5 ~~ s200_af.6
          s51_01.4 ~~ s51_04.4
          s51_01.4 ~~ s51_07.4
          s51_01.4 ~~ s51_09.4
          s51_04.4 ~~ s51_07.4
          s51_04.4 ~~ s51_09.4
          s51_07.4 ~~ s51_09.4
          s51_01.5 ~~ s51_04.5
          s51_01.5 ~~ s51_07.5
          s51_01.5 ~~ s51_09.5
          s51_04.5 ~~ s51_07.5
          s51_04.5 ~~ s51_09.5
          s51_07.5 ~~ s51_09.5
          s51_01.6 ~~ s51_04.6
          s51_01.6 ~~ s51_07.6
          s51_01.6 ~~ s51_09.6
          s51_04.6 ~~ s51_07.6
          s51_04.6 ~~ s51_09.6
          s51_07.6 ~~ s51_09.6
          s51_01.4 ~~ s51_01.5 + s51_01.6
          s51_01.5 ~~ s51_01.6
          s51_02.4 ~~ s51_02.5 + s51_02.6
          s51_02.5 ~~ s51_02.6
          s51_03.4 ~~ s51_03.5 + s51_03.6
          s51_03.5 ~~ s51_03.6
          s51_04.4 ~~ s51_04.5 + s51_04.6
          s51_04.5 ~~ s51_04.6
          s51_05.4 ~~ s51_05.5 + s51_05.6
          s51_05.5 ~~ s51_05.6
          s51_06.4 ~~ s51_06.5 + s51_06.6
          s51_06.5 ~~ s51_06.6
          s51_07.4 ~~ s51_07.5 + s51_07.6
          s51_07.5 ~~ s51_07.6
          s51_08.4 ~~ s51_08.5 + s51_08.6
          s51_08.5 ~~ s51_08.6
          s51_09.4 ~~ s51_09.5 + s51_09.6
          s51_09.5 ~~ s51_09.6
'

#  Model estimation
```

```
fit4 <- sem(model1,
            data = df,
            estimator = "ML",
            missing = "ML")

summary(fit4,
        rsquare = TRUE,
        standardized = TRUE,
        fit.measures = TRUE)
```

Alternative model IV: Without path SOC -> MWB

```
model1 <- '
          # Defining latent variables
          WB.1 =~ a1*s91_01.4 + a2*s91_02.4 + a3*s91_03.4 + a4*s91_04.4 + a5*s91_05.4 + a6*s91_06.4 + a7
          WB.2 =~ a1*s91_01.5 + a2*s91_02.5 + a3*s91_03.5 + a4*s91_04.5 + a5*s91_05.5 + a6*s91_06.5 + a7
          WB.3 =~ a1*s91_01.6 + a2*s91_02.6 + a3*s91_03.6 + a4*s91_04.6 + a5*s91_05.6 + a6*s91_06.6 + a7
          SoC.1 =~ b1*s51_01.4 + b2*s51_02.4 + b3*s51_03.4 + b4*s51_04.4 + b5*s51_05.4 + b6*s51_06.4 + b
          SoC.2 =~ b1*s51_01.5 + b2*s51_02.5 + b3*s51_03.5 + b4*s51_04.5 + b5*s51_05.5 + b6*s51_06.5 + b
          SoC.3 =~ b1*s51_01.6 + b2*s51_02.6 + b3*s51_03.6 + b4*s51_04.6 + b5*s51_05.6 + b6*s51_06.6 + b
          OJC.1 =~ c1*s200_de.4 + c2*s200_re.4 + c3*s200_au.4 + c4*s200_me.4 + c5*s200_ma.4 + c6*s200_ar
          OJC.2 =~ c1*s200_de.5 + c2*s200_re.5 + c3*s200_au.5 + c4*s200_me.5 + c5*s200_ma.5 + c6*s200_ar
          OJC.3 =~ c1*s200_de.6 + c2*s200_re.6 + c3*s200_au.6 + c4*s200_me.6 + c5*s200_ma.6 + c6*s200_ar

          # Structural model
          SoC.2 ~ SoC.1 + a*OJC.1
          SoC.3 ~ SoC.2 + a*OJC.2
          OJC.2 ~ OJC.1 + b*SoC.1
          OJC.3 ~ OJC.2 + b*SoC.2
          WB.2 ~ WB.1 + d*OJC.1
          WB.3 ~ WB.2 + d*OJC.2

          # Covariances
          SoC.1 ~~ OJC.1 + WB.1
          OJC.1 ~~ WB.1
          SoC.2 ~~ OJC.2 + WB.2
          OJC.2 ~~ WB.2
          SoC.3 ~~ OJC.3 + WB.3
          OJC.3 ~~ WB.3

          # Residual covariances
          s91_01.4 ~~ s91_02.4
          s91_02.4 ~~ s91_06.4
          s91_04.4 ~~ s91_05.4
          s91_04.4 ~~ s91_07.4
          s91_05.4 ~~ s91_07.4
          s91_01.5 ~~ s91_02.5
          s91_02.5 ~~ s91_06.5
          s91_04.5 ~~ s91_05.5
          s91_04.5 ~~ s91_07.5
          s91_05.5 ~~ s91_07.5
          s91_01.6 ~~ s91_02.6
          s91_02.6 ~~ s91_06.6
```

```
s91_04.6 ~~ s91_05.6
s91_04.6 ~~ s91_07.6
s91_05.6 ~~ s91_07.6
s91_01.4 ~~ s91_01.5 + s91_01.6
s91_01.5 ~~ s91_01.6
s91_02.4 ~~ s91_02.5 + s91_02.6
s91_02.5 ~~ s91_02.6
s91_03.4 ~~ s91_03.5 + s91_03.6
s91_03.5 ~~ s91_03.6
s91_04.4 ~~ s91_04.5 + s91_04.6
s91_04.5 ~~ s91_04.6
s91_05.4 ~~ s91_05.5 + s91_05.6
s91_05.5 ~~ s91_05.6
s91_06.4 ~~ s91_06.5 + s91_06.6
s91_06.5 ~~ s91_06.6
s91_07.4 ~~ s91_07.5 + s91_07.6
s91_07.5 ~~ s91_07.6
s200_de.4 ~~ s200_re.4
s200_de.5 ~~ s200_re.5
s200_de.6 ~~ s200_re.6
s200_me.4 ~~ s200_af.4
s200_me.5 ~~ s200_af.5
s200_me.6 ~~ s200_af.6
s200_de.4 ~~ s200_de.5 + s200_de.6
s200_de.5 ~~ s200_de.6
s200_re.4 ~~ s200_re.5 + s200_re.6
s200_re.5 ~~ s200_re.6
s200_au.4 ~~ s200_au.5 + s200_au.6
s200_au.5 ~~ s200_au.6
s200_me.4 ~~ s200_me.5 + s200_me.6
s200_me.5 ~~ s200_me.6
s200_ma.4 ~~ s200_ma.5 + s200_ma.6
s200_ma.5 ~~ s200_ma.6
s200_af.4 ~~ s200_af.5 + s200_af.6
s200_af.5 ~~ s200_af.6
s51_01.4 ~~ s51_04.4
s51_01.4 ~~ s51_07.4
s51_01.4 ~~ s51_09.4
s51_04.4 ~~ s51_07.4
s51_04.4 ~~ s51_09.4
s51_07.4 ~~ s51_09.4
s51_01.5 ~~ s51_04.5
s51_01.5 ~~ s51_07.5
s51_01.5 ~~ s51_09.5
s51_04.5 ~~ s51_07.5
s51_04.5 ~~ s51_09.5
s51_07.5 ~~ s51_09.5
s51_01.6 ~~ s51_04.6
s51_01.6 ~~ s51_07.6
s51_01.6 ~~ s51_09.6
s51_04.6 ~~ s51_07.6
s51_04.6 ~~ s51_09.6
s51_07.6 ~~ s51_09.6
```

```
          s51_01.4 ~~ s51_01.5 + s51_01.6
          s51_01.5 ~~ s51_01.6
          s51_02.4 ~~ s51_02.5 + s51_02.6
          s51_02.5 ~~ s51_02.6
          s51_03.4 ~~ s51_03.5 + s51_03.6
          s51_03.5 ~~ s51_03.6
          s51_04.4 ~~ s51_04.5 + s51_04.6
          s51_04.5 ~~ s51_04.6
          s51_05.4 ~~ s51_05.5 + s51_05.6
          s51_05.5 ~~ s51_05.6
          s51_06.4 ~~ s51_06.5 + s51_06.6
          s51_06.5 ~~ s51_06.6
          s51_07.4 ~~ s51_07.5 + s51_07.6
          s51_07.5 ~~ s51_07.6
          s51_08.4 ~~ s51_08.5 + s51_08.6
          s51_08.5 ~~ s51_08.6
          s51_09.4 ~~ s51_09.5 + s51_09.6
          s51_09.5 ~~ s51_09.6
'

#  Model estimation
fit5 <- sem(model1,
            data = df,
            estimator = "ML",
            missing = "ML")

summary(fit5,
        rsquare = TRUE,
        standardized = TRUE,
        fit.measures = TRUE)
```

Alternative model V: Additional path MWB -> SOC

```
model1 <- '
          # Defining latent variables
          WB.1 =~ a1*s91_01.4 + a2*s91_02.4 + a3*s91_03.4 + a4*s91_04.4 + a5*s91_05.4 + a6*s91_06.4 + a
          WB.2 =~ a1*s91_01.5 + a2*s91_02.5 + a3*s91_03.5 + a4*s91_04.5 + a5*s91_05.5 + a6*s91_06.5 + a
          WB.3 =~ a1*s91_01.6 + a2*s91_02.6 + a3*s91_03.6 + a4*s91_04.6 + a5*s91_05.6 + a6*s91_06.6 + a
          SoC.1 =~ b1*s51_01.4 + b2*s51_02.4 + b3*s51_03.4 + b4*s51_04.4 + b5*s51_05.4 + b6*s51_06.4 +
          SoC.2 =~ b1*s51_01.5 + b2*s51_02.5 + b3*s51_03.5 + b4*s51_04.5 + b5*s51_05.5 + b6*s51_06.5 +
          SoC.3 =~ b1*s51_01.6 + b2*s51_02.6 + b3*s51_03.6 + b4*s51_04.6 + b5*s51_05.6 + b6*s51_06.6 +
          OJC.1 =~ c1*s200_de.4 + c2*s200_re.4 + c3*s200_au.4 + c4*s200_me.4 + c5*s200_ma.4 + c6*s200_a
          OJC.2 =~ c1*s200_de.5 + c2*s200_re.5 + c3*s200_au.5 + c4*s200_me.5 + c5*s200_ma.5 + c6*s200_a
          OJC.3 =~ c1*s200_de.6 + c2*s200_re.6 + c3*s200_au.6 + c4*s200_me.6 + c5*s200_ma.6 + c6*s200_a

          # Structural model
          SoC.2 ~ SoC.1 + a*OJC.1 + b*WB.1
          SoC.3 ~ SoC.2 + a*OJC.2 + b*WB.2
          OJC.2 ~ OJC.1 + c*SoC.1
          OJC.3 ~ OJC.2 + c*SoC.2
          WB.2 ~ WB.1 + d*OJC.1 + e*SoC.1
          WB.3 ~ WB.2 + d*OJC.2 + e*SoC.2
```

```
# Covariances
SoC.1 ~~ OJC.1 + WB.1
OJC.1 ~~ WB.1
SoC.2 ~~ OJC.2 + WB.2
OJC.2 ~~ WB.2
SoC.3 ~~ OJC.3 + WB.3
OJC.3 ~~ WB.3

# Residual covariances
s91_01.4 ~~ s91_02.4
s91_02.4 ~~ s91_06.4
s91_04.4 ~~ s91_05.4
s91_04.4 ~~ s91_07.4
s91_05.4 ~~ s91_07.4
s91_01.5 ~~ s91_02.5
s91_02.5 ~~ s91_06.5
s91_04.5 ~~ s91_05.5
s91_04.5 ~~ s91_07.5
s91_05.5 ~~ s91_07.5
s91_01.6 ~~ s91_02.6
s91_02.6 ~~ s91_06.6
s91_04.6 ~~ s91_05.6
s91_04.6 ~~ s91_07.6
s91_05.6 ~~ s91_07.6
s91_01.4 ~~ s91_01.5 + s91_01.6
s91_01.5 ~~ s91_01.6
s91_02.4 ~~ s91_02.5 + s91_02.6
s91_02.5 ~~ s91_02.6
s91_03.4 ~~ s91_03.5 + s91_03.6
s91_03.5 ~~ s91_03.6
s91_04.4 ~~ s91_04.5 + s91_04.6
s91_04.5 ~~ s91_04.6
s91_05.4 ~~ s91_05.5 + s91_05.6
s91_05.5 ~~ s91_05.6
s91_06.4 ~~ s91_06.5 + s91_06.6
s91_06.5 ~~ s91_06.6
s91_07.4 ~~ s91_07.5 + s91_07.6
s91_07.5 ~~ s91_07.6
s200_de.4 ~~ s200_re.4
s200_de.5 ~~ s200_re.5
s200_de.6 ~~ s200_re.6
s200_me.4 ~~ s200_af.4
s200_me.5 ~~ s200_af.5
s200_me.6 ~~ s200_af.6
s200_de.4 ~~ s200_de.5 + s200_de.6
s200_de.5 ~~ s200_de.6
s200_re.4 ~~ s200_re.5 + s200_re.6
s200_re.5 ~~ s200_re.6
s200_au.4 ~~ s200_au.5 + s200_au.6
s200_au.5 ~~ s200_au.6
s200_me.4 ~~ s200_me.5 + s200_me.6
s200_me.5 ~~ s200_me.6
s200_ma.4 ~~ s200_ma.5 + s200_ma.6
```

```
           s200_ma.5 ~~ s200_ma.6
           s200_af.4 ~~ s200_af.5 + s200_af.6
           s200_af.5 ~~ s200_af.6
           s51_01.4 ~~ s51_04.4
           s51_01.4 ~~ s51_07.4
           s51_01.4 ~~ s51_09.4
           s51_04.4 ~~ s51_07.4
           s51_04.4 ~~ s51_09.4
           s51_07.4 ~~ s51_09.4
           s51_01.5 ~~ s51_04.5
           s51_01.5 ~~ s51_07.5
           s51_01.5 ~~ s51_09.5
           s51_04.5 ~~ s51_07.5
           s51_04.5 ~~ s51_09.5
           s51_07.5 ~~ s51_09.5
           s51_01.6 ~~ s51_04.6
           s51_01.6 ~~ s51_07.6
           s51_01.6 ~~ s51_09.6
           s51_04.6 ~~ s51_07.6
           s51_04.6 ~~ s51_09.6
           s51_07.6 ~~ s51_09.6
           s51_01.4 ~~ s51_01.5 + s51_01.6
           s51_01.5 ~~ s51_01.6
           s51_02.4 ~~ s51_02.5 + s51_02.6
           s51_02.5 ~~ s51_02.6
           s51_03.4 ~~ s51_03.5 + s51_03.6
           s51_03.5 ~~ s51_03.6
           s51_04.4 ~~ s51_04.5 + s51_04.6
           s51_04.5 ~~ s51_04.6
           s51_05.4 ~~ s51_05.5 + s51_05.6
           s51_05.5 ~~ s51_05.6
           s51_06.4 ~~ s51_06.5 + s51_06.6
           s51_06.5 ~~ s51_06.6
           s51_07.4 ~~ s51_07.5 + s51_07.6
           s51_07.5 ~~ s51_07.6
           s51_08.4 ~~ s51_08.5 + s51_08.6
           s51_08.5 ~~ s51_08.6
           s51_09.4 ~~ s51_09.5 + s51_09.6
           s51_09.5 ~~ s51_09.6
'

#  Model estimation
fit6 <- sem(model1,
            data = df,
            estimator = "ML",
            missing = "ML")

summary(fit6,
        rsquare = TRUE,
        standardized = TRUE,
        fit.measures = TRUE)
```

Alternative model VI: Additional path MWB -> OJC

```
model1 <- '
          # Defining latent variables
          WB.1 =~ a1*s91_01.4 + a2*s91_02.4 + a3*s91_03.4 + a4*s91_04.4 + a5*s91_05.4 + a6*s91_06.4 + a
          WB.2 =~ a1*s91_01.5 + a2*s91_02.5 + a3*s91_03.5 + a4*s91_04.5 + a5*s91_05.5 + a6*s91_06.5 + a
          WB.3 =~ a1*s91_01.6 + a2*s91_02.6 + a3*s91_03.6 + a4*s91_04.6 + a5*s91_05.6 + a6*s91_06.6 + a
          SoC.1 =~ b1*s51_01.4 + b2*s51_02.4 + b3*s51_03.4 + b4*s51_04.4 + b5*s51_05.4 + b6*s51_06.4 +
          SoC.2 =~ b1*s51_01.5 + b2*s51_02.5 + b3*s51_03.5 + b4*s51_04.5 + b5*s51_05.5 + b6*s51_06.5 +
          SoC.3 =~ b1*s51_01.6 + b2*s51_02.6 + b3*s51_03.6 + b4*s51_04.6 + b5*s51_05.6 + b6*s51_06.6 +
          OJC.1 =~ c1*s200_de.4 + c2*s200_re.4 + c3*s200_au.4 + c4*s200_me.4 + c5*s200_ma.4 + c6*s200_a
          OJC.2 =~ c1*s200_de.5 + c2*s200_re.5 + c3*s200_au.5 + c4*s200_me.5 + c5*s200_ma.5 + c6*s200_a
          OJC.3 =~ c1*s200_de.6 + c2*s200_re.6 + c3*s200_au.6 + c4*s200_me.6 + c5*s200_ma.6 + c6*s200_a

          # Structural model
          SoC.2 ~ SoC.1 + a*OJC.1
          SoC.3 ~ SoC.2 + a*OJC.2
          OJC.2 ~ OJC.1 + b*SoC.1 + c*WB.1
          OJC.3 ~ OJC.2 + b*SoC.2 + c*WB.2
          WB.2 ~ WB.1 + d*OJC.1 + e*SoC.1
          WB.3 ~ WB.2 + d*OJC.2 + e*SoC.2

          # Covariances
          SoC.1 ~~ OJC.1 + WB.1
          OJC.1 ~~ WB.1
          SoC.2 ~~ OJC.2 + WB.2
          OJC.2 ~~ WB.2
          SoC.3 ~~ OJC.3 + WB.3
          OJC.3 ~~ WB.3

          # Residual covariances
          s91_01.4 ~~ s91_02.4
          s91_02.4 ~~ s91_06.4
          s91_04.4 ~~ s91_05.4
          s91_04.4 ~~ s91_07.4
          s91_05.4 ~~ s91_07.4
          s91_01.5 ~~ s91_02.5
          s91_02.5 ~~ s91_06.5
          s91_04.5 ~~ s91_05.5
          s91_04.5 ~~ s91_07.5
          s91_05.5 ~~ s91_07.5
          s91_01.6 ~~ s91_02.6
          s91_02.6 ~~ s91_06.6
          s91_04.6 ~~ s91_05.6
          s91_04.6 ~~ s91_07.6
          s91_05.6 ~~ s91_07.6
          s91_01.4 ~~ s91_01.5 + s91_01.6
          s91_01.5 ~~ s91_01.6
          s91_02.4 ~~ s91_02.5 + s91_02.6
          s91_02.5 ~~ s91_02.6
          s91_03.4 ~~ s91_03.5 + s91_03.6
          s91_03.5 ~~ s91_03.6
          s91_04.4 ~~ s91_04.5 + s91_04.6
          s91_04.5 ~~ s91_04.6
          s91_05.4 ~~ s91_05.5 + s91_05.6
```

```
s91_05.5 ~~ s91_05.6
s91_06.4 ~~ s91_06.5 + s91_06.6
s91_06.5 ~~ s91_06.6
s91_07.4 ~~ s91_07.5 + s91_07.6
s91_07.5 ~~ s91_07.6
s200_de.4 ~~ s200_re.4
s200_de.5 ~~ s200_re.5
s200_de.6 ~~ s200_re.6
s200_me.4 ~~ s200_af.4
s200_me.5 ~~ s200_af.5
s200_me.6 ~~ s200_af.6
s200_de.4 ~~ s200_de.5 + s200_de.6
s200_de.5 ~~ s200_de.6
s200_re.4 ~~ s200_re.5 + s200_re.6
s200_re.5 ~~ s200_re.6
s200_au.4 ~~ s200_au.5 + s200_au.6
s200_au.5 ~~ s200_au.6
s200_me.4 ~~ s200_me.5 + s200_me.6
s200_me.5 ~~ s200_me.6
s200_ma.4 ~~ s200_ma.5 + s200_ma.6
s200_ma.5 ~~ s200_ma.6
s200_af.4 ~~ s200_af.5 + s200_af.6
s200_af.5 ~~ s200_af.6
s51_01.4 ~~ s51_04.4
s51_01.4 ~~ s51_07.4
s51_01.4 ~~ s51_09.4
s51_04.4 ~~ s51_07.4
s51_04.4 ~~ s51_09.4
s51_07.4 ~~ s51_09.4
s51_01.5 ~~ s51_04.5
s51_01.5 ~~ s51_07.5
s51_01.5 ~~ s51_09.5
s51_04.5 ~~ s51_07.5
s51_04.5 ~~ s51_09.5
s51_07.5 ~~ s51_09.5
s51_01.6 ~~ s51_04.6
s51_01.6 ~~ s51_07.6
s51_01.6 ~~ s51_09.6
s51_04.6 ~~ s51_07.6
s51_04.6 ~~ s51_09.6
s51_07.6 ~~ s51_09.6
s51_01.4 ~~ s51_01.5 + s51_01.6
s51_01.5 ~~ s51_01.6
s51_02.4 ~~ s51_02.5 + s51_02.6
s51_02.5 ~~ s51_02.6
s51_03.4 ~~ s51_03.5 + s51_03.6
s51_03.5 ~~ s51_03.6
s51_04.4 ~~ s51_04.5 + s51_04.6
s51_04.5 ~~ s51_04.6
s51_05.4 ~~ s51_05.5 + s51_05.6
s51_05.5 ~~ s51_05.6
s51_06.4 ~~ s51_06.5 + s51_06.6
s51_06.5 ~~ s51_06.6
```

```
          s51_07.4 ~~ s51_07.5 + s51_07.6
          s51_07.5 ~~ s51_07.6
          s51_08.4 ~~ s51_08.5 + s51_08.6
          s51_08.5 ~~ s51_08.6
          s51_09.4 ~~ s51_09.5 + s51_09.6
          s51_09.5 ~~ s51_09.6
'

#  Model estimation
fit7 <- sem(model1,
            data = df,
            estimator = "ML",
            missing = "ML")

summary(fit7,
        rsquare = TRUE,
        standardized = TRUE,
        fit.measures = TRUE)
```

Final model

```
model1 <- '
          # Defining latent variables
          WB.1 =~ a1*s91_01.4 + a2*s91_02.4 + a3*s91_03.4 + a4*s91_04.4 + a5*s91_05.4 + a6*s91_06.4 + a7
          WB.2 =~ a1*s91_01.5 + a2*s91_02.5 + a3*s91_03.5 + a4*s91_04.5 + a5*s91_05.5 + a6*s91_06.5 + a7
          WB.3 =~ a1*s91_01.6 + a2*s91_02.6 + a3*s91_03.6 + a4*s91_04.6 + a5*s91_05.6 + a6*s91_06.6 + a7
          SoC.1 =~ b1*s51_01.4 + b2*s51_02.4 + b3*s51_03.4 + b4*s51_04.4 + b5*s51_05.4 + b6*s51_06.4 + b
          SoC.2 =~ b1*s51_01.5 + b2*s51_02.5 + b3*s51_03.5 + b4*s51_04.5 + b5*s51_05.5 + b6*s51_06.5 + b
          SoC.3 =~ b1*s51_01.6 + b2*s51_02.6 + b3*s51_03.6 + b4*s51_04.6 + b5*s51_05.6 + b6*s51_06.6 + b
          OJC.1 =~ c1*s200_de.4 + c2*s200_re.4 + c3*s200_au.4 + c4*s200_me.4 + c5*s200_ma.4 + c6*s200_a
          OJC.2 =~ c1*s200_de.5 + c2*s200_re.5 + c3*s200_au.5 + c4*s200_me.5 + c5*s200_ma.5 + c6*s200_a
          OJC.3 =~ c1*s200_de.6 + c2*s200_re.6 + c3*s200_au.6 + c4*s200_me.6 + c5*s200_ma.6 + c6*s200_a

          # Structural model
          SoC.2 ~ SoC.1 + a*WB.1
          SoC.3 ~ SoC.2 + a*WB.2
          OJC.2 ~ OJC.1 + b*SoC.1 + c*WB.1
          OJC.3 ~ OJC.2 + b*SoC.2 + c*WB.2
          WB.2 ~ WB.1 + d*OJC.1 + e*SoC.1
          WB.3 ~ WB.2 + d*OJC.2 + e*SoC.2

          # Covariances
          SoC.1 ~~ OJC.1 + WB.1
          OJC.1 ~~ WB.1
          SoC.2 ~~ OJC.2 + WB.2
          OJC.2 ~~ WB.2
          SoC.3 ~~ OJC.3 + WB.3
          OJC.3 ~~ WB.3

          # Residual covariances
          s91_01.4 ~~ s91_02.4
          s91_02.4 ~~ s91_06.4
          s91_04.4 ~~ s91_05.4
```

```
s91_04.4 ~~ s91_07.4
s91_05.4 ~~ s91_07.4
s91_01.5 ~~ s91_02.5
s91_02.5 ~~ s91_06.5
s91_04.5 ~~ s91_05.5
s91_04.5 ~~ s91_07.5
s91_05.5 ~~ s91_07.5
s91_01.6 ~~ s91_02.6
s91_02.6 ~~ s91_06.6
s91_04.6 ~~ s91_05.6
s91_04.6 ~~ s91_07.6
s91_05.6 ~~ s91_07.6
s91_01.4 ~~ s91_01.5 + s91_01.6
s91_01.5 ~~ s91_01.6
s91_02.4 ~~ s91_02.5 + s91_02.6
s91_02.5 ~~ s91_02.6
s91_03.4 ~~ s91_03.5 + s91_03.6
s91_03.5 ~~ s91_03.6
s91_04.4 ~~ s91_04.5 + s91_04.6
s91_04.5 ~~ s91_04.6
s91_05.4 ~~ s91_05.5 + s91_05.6
s91_05.5 ~~ s91_05.6
s91_06.4 ~~ s91_06.5 + s91_06.6
s91_06.5 ~~ s91_06.6
s91_07.4 ~~ s91_07.5 + s91_07.6
s91_07.5 ~~ s91_07.6
s200_de.4 ~~ s200_re.4
s200_de.5 ~~ s200_re.5
s200_de.6 ~~ s200_re.6
s200_me.4 ~~ s200_af.4
s200_me.5 ~~ s200_af.5
s200_me.6 ~~ s200_af.6
s200_de.4 ~~ s200_de.5 + s200_de.6
s200_de.5 ~~ s200_de.6
s200_re.4 ~~ s200_re.5 + s200_re.6
s200_re.5 ~~ s200_re.6
s200_au.4 ~~ s200_au.5 + s200_au.6
s200_au.5 ~~ s200_au.6
s200_me.4 ~~ s200_me.5 + s200_me.6
s200_me.5 ~~ s200_me.6
s200_ma.4 ~~ s200_ma.5 + s200_ma.6
s200_ma.5 ~~ s200_ma.6
s200_af.4 ~~ s200_af.5 + s200_af.6
s200_af.5 ~~ s200_af.6
s51_01.4 ~~ s51_04.4
s51_01.4 ~~ s51_07.4
s51_01.4 ~~ s51_09.4
s51_04.4 ~~ s51_07.4
s51_04.4 ~~ s51_09.4
s51_07.4 ~~ s51_09.4
s51_01.5 ~~ s51_04.5
s51_01.5 ~~ s51_07.5
s51_01.5 ~~ s51_09.5
```

```
          s51_04.5 ~~ s51_07.5
          s51_04.5 ~~ s51_09.5
          s51_07.5 ~~ s51_09.5
          s51_01.6 ~~ s51_04.6
          s51_01.6 ~~ s51_07.6
          s51_01.6 ~~ s51_09.6
          s51_04.6 ~~ s51_07.6
          s51_04.6 ~~ s51_09.6
          s51_07.6 ~~ s51_09.6
          s51_01.4 ~~ s51_01.5 + s51_01.6
          s51_01.5 ~~ s51_01.6
          s51_02.4 ~~ s51_02.5 + s51_02.6
          s51_02.5 ~~ s51_02.6
          s51_03.4 ~~ s51_03.5 + s51_03.6
          s51_03.5 ~~ s51_03.6
          s51_04.4 ~~ s51_04.5 + s51_04.6
          s51_04.5 ~~ s51_04.6
          s51_05.4 ~~ s51_05.5 + s51_05.6
          s51_05.5 ~~ s51_05.6
          s51_06.4 ~~ s51_06.5 + s51_06.6
          s51_06.5 ~~ s51_06.6
          s51_07.4 ~~ s51_07.5 + s51_07.6
          s51_07.5 ~~ s51_07.6
          s51_08.4 ~~ s51_08.5 + s51_08.6
          s51_08.5 ~~ s51_08.6
          s51_09.4 ~~ s51_09.5 + s51_09.6
          s51_09.5 ~~ s51_09.6
'

#  Model estimation
fit8 <- sem(model1,
            data = df,
            estimator = "ML",
            missing = "ML")

summary(fit8,
        rsquare = TRUE,
        standardized = TRUE,
        fit.measures = TRUE)
```

Test of invariance and model comparisons

```
anova(fit0a, fit0b)
anova(fit1, fit2)
anova(fit1, fit3)
anova(fit1, fit4)
anova(fit1, fit5)
anova(fit1, fit6)
anova(fit1, fit7)
anova(fit1, fit8)
```
